# Iron-Related Parameters are Altered Between C57BL/6N and C57BL/6J *Mus Musculus* Wild-Type Substrains

**DOI:** 10.1097/HS9.0000000000000304

**Published:** 2019-11-09

**Authors:** Oriana Marques, Joana Neves, Natalie K. Horvat, Silvia Colucci, Claudia Guida, Martina U. Muckenthaler

**Affiliations:** 1 Department of Pediatric Oncology, Hematology and Immunology, University Hospital Heidelberg, Heidelberg, Germany; 2 Molecular Medicine Partnership Unit, University of Heidelberg, Heidelberg, Germany; 3 European Molecular Biology Laboratory, Heidelberg, Germany.

*Mus musculus* BL6 strains, specifically C57BL/6N and C57BL/6J, are frequently analyzed in an interchangeable manner as ‘wildtype’ controls without indication of the substrain. We analyzed hematological and iron-related parameters in C57BL/6N and C57BL/6J mice and show differences in the hematocrit, the mean corpuscular volume as well as the non-heme iron content of the spleen, which was significantly higher in the C57BL/6J compared to the C57BL/6N substrain. At the molecular level, elevated splenic iron levels were associated with higher ferritin (FTL and FTH) and lower transferrin receptor 1 (TfR1) protein levels. A sequence variation in Herc2, an E3 ubiquitin ligase that promotes Nuclear Receptor Coactivator 4 (NCOA4) ubiquitination and degradation, which in turn mediates the autophagic turnover of ferritin, may offer an explanation for the observed phenotype.

Inbred mouse lines have been essential for biomedical research. They allowed for the execution of reproducible experiments on identical genetic material, in different research centers across the world.^1,2^ In theory, within inbred mouse strains, each and every mouse should share the same allele for every DNA sequence, thus being genetically equal. However, genetic fixation is not stable across time, due to the fact that a residual proportion of mice may still be heterozygous for specific *loci* during inbreeding and that spontaneous mutations may introduce *de novo* heterozygosity.^3^ Thus, genetic drift from the original inbred strain may generate new substrains.^2^

In the 1920s, C. C. Little established the C57BL/6 line, which rapidly became the most frequently used genetic background to analyze spontaneous and induced mutations.^4,5^ The two major C57BL/6 substrains are known as C57BL/6J and C57BL/6N. C57BL/6J is the original Jackson Laboratory mouse strain derived from the original C57BL/6 stock from C. C. Little. Later, in 1951, as a result of the separation from the C57BL/6J, the C57BL/6N substrain arose at the National Institutes of Health. In the literature, these substrains are commonly treated as equal and are referred to as “C57” or “B6”. Recent assessment of the genetic variation between the C57BL/6J and C57BL/6N substrains revealed 34 single-nucleotide polymorphisms (SNPs) and 2 indels distinguishing coding sequences, as well as 15 structural variants, such as products of retrotransposition or variable number tandem repeats, overlapping genes.^6^ Therefore, it is not surprising that reports describe phenotypic differences between these 2 substrains, including behavior, glucose and hormonal homeostasis, alcohol intake and preference and drug influence (reviewed in ^3,7^). The continuous lack of appreciation for the existence of different substrains will lead to mixed or uncertain C57BL/6 backgrounds that must be avoided if one wants to correctly interpret genetic and phenotypical analyses.

Given the reported genetic variations we sought to elucidate the hematological and iron-related differences between C57BL/6J and C57BL/6N substrains. We compared serum iron concentration and hematological parameters in 12-week-old male C57BL/6N and C57BL/6J mice (Table 1). Serum iron levels, unsaturated iron binding capacity (UIBC), transferrin saturation (TfSat) and hemoglobin (Hb) content were not significantly different. By contrast, the hematocrit (Hct) was significantly increased in C57BL/6J mice, likely reflecting the mild increase in red blood cell counts (RBC) and the enlarged mean corpuscular volume (MCV) in the C57BL/6J substrain. These phenotype differences are in line with reports from the European Mouse Disease Clinic consortium.^6^

**Table 1:**
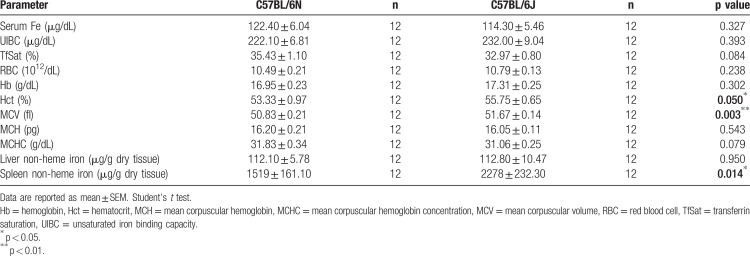
Serum and Tissue Iron Levels and Hematological Parameters in 12-Week-Old Male Mice.

Due to the important role of the liver in controlling systemic iron homeostasis and of splenic macrophages in recycling hemoglobin derived iron from aged red blood cells, we also compared liver and spleen non-heme iron content between C57BL/6N and C57BL/6J mice. In the liver we did not detect differences in the iron content as assessed by the bathophenanthroline method (Table 1) and DAB-enhanced Perls’ staining (SDC Fig. 1A, Supplemental Digital Content). Likewise, mRNA expression of the iron-controlled hormone hepcidin (SDC Figure 1b, Supplemental Digital Content) responsible for regulating systemic iron levels, as well as transferrin receptor 1 (TFR1), ferritin light chain (FTL), ferritin heavy chain (FTH) and ferroportin (Fpn) mRNA and protein levels (Fig. 1A–D; SDC Fig. 1C-H, Supplemental Digital Content) remained unaltered.

**Figure 1:**
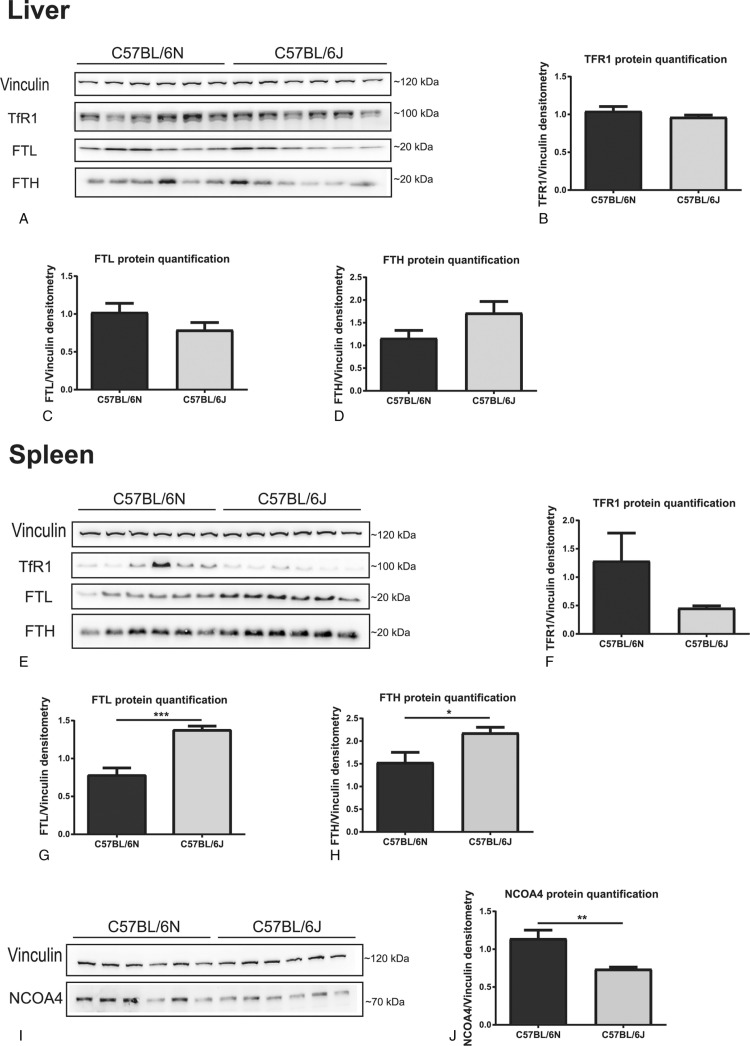
**C57BL/6J mice show increased spleen, but not liver, ferritin levels, in comparison with C57BL/6N mice**. Liver: (A–D) Western-blot analysis (n = 6) of hepatic TFR1 (A,B), FTL (A,C) and FTH (A,D). Spleen: (E-F) Western-blot analysis (n = 6) of splenic TFR1 (E,F), FTL (E,G) and FTH (E,H). (I,J) Western-blot analysis (n = 6) of splenic NCOA4. Vinculin was used as a loading control for western-blots. One representative loading control is shown. Data are reported as mean ± SEM. Student's *t* test: ^∗^p < 0.05; ^∗∗^p < 0.01; ^∗∗∗^p < 0.001.

In contrast, the splenic non-heme iron content was strongly increased in C57BL/6J mice (Table 1), a finding confirmed by histological analysis with DAB-enhanced Perls’ staining with iron deposits predominantly occurring in red pulp macrophages (SDC Fig. 1I, Supplemental Digital Content). At the molecular level, the higher splenic iron content in C57BL/6J mice was correlated with higher FTL and FTH protein levels and a trend towards lower TFR1 protein levels (Fig. 1E–H). These gene responses are consistent with the post-transcriptional regulation by the iron-responsive element/iron-regulatory protein (IRE/IRP) system, with the expected inhibition of IRP activity due to high iron levels,^8^ and unaltered *TfR1*, *Ftl1* and *Fth1* mRNA levels (SDC Figure 1j-l, Supplemental Digital Content). Consistent with unaltered hepatic hepcidin expression, mRNA and protein levels of the iron exporter ferroportin (FPN) were not significantly different between these two mouse substrains and therefore cannot explain the higher splenic iron amounts in C57BL/6J mice (SDC Figure 1M,N, Supplemental Digital Content). Moreover, spleen weights were similar between C57BL/6N and C57BL/6J mice (data not shown).

A recent study by Simon et al reported a detailed genomic and phenotypic comparison between C57BL/6J and C57BL/6N mouse strains, describing a total of 51 genes with sequence or structural variants in coding sequences between substrains. One SNP was identified in *Bmpr2, a gene* associated with hepcidin transcriptional control in hepatocytes.^9^ However, the 2 substrains did not differ in hepatic *hepcidin* mRNA levels (SDC Fig. 1B, Supplemental Digital Content). We next studied a putative role of *Bmpr2* in maintaining macrophage iron levels. While RNAi for *Bmpr2* successfully decreased its mRNA expression in Bone Marrow-derived Macrophages (BMDMs) (SDC Fig. 2A, Supplemental Digital Content) it did not significantly alter *Fth* and *Ftl* mRNA and protein levels, suggesting that the iron content remained unaffected (SDC B–G, Supplemental Digital Content), thus excluding the involvement of the *Bmpr2* SNP in explaining changes in splenic iron levels.

Another sequence variation between the C57BL/6J and C57BL/6N substrains was identified in *Herc2*, an E3 ubiquitin ligase that promotes NCOA4 ubiquitination and degradation in an iron-dependent manner.^10^ NCOA4 is a cargo receptor that mediates the autophagic turnover of ferritin,^11,12^ preventing iron accumulation and guaranteeing efficient erythropoiesis.^13^*In silico* modeling and mutagenesis of the HERC2 cytochrome b5 heme-binding subdomain structure showed that the amino acid exchange in HERC2 (G1235D; C57BL/6J-G; C57BL/6N-D) does not appear to change the secondary structure of this subdomain (SDC Fig. 3B, Supplemental Digital Content), but affects the overall surface charge (SDC Fig. 3C, Supplemental Digital Content) that is expected to have implications on protein stability and interactions. Moreover, the aminoacid in position 1235 of the HERC2 cytochrome b5 heme-binding subdomain is highly conserved among species, with only C57BL/6J mice encoding glycine instead of aspartic acid (SDC Fig. 3D, Supplemental Digital Content), suggesting that this residue may be critical for protein function. Sanger sequencing confirmed that the C57BL/6N and C57BL/6J colonies analyzed here differ at the HERC2 SNP previously identified by Simon and collaborators (data not shown). Consistently, splenic NCOA4 protein (Fig. 1I,J), and tendentially mRNA levels (SDC Fig. 1P, Supplemental Digital Content) are reduced in C57BL/6J mice, in line with increased ferritin levels (Fig. 1E,G,H). Thus, altered HERC2 activity could impact on NCOA4 protein levels and provide an explanation for the increased ferritin levels observed in the spleen of C57BL/6J mice. These results are in agreement with previous observations that *Ncoa4*^−/−^ mice present an increase in ferritin and iron in splenic macrophages, despite liver and duodenum normal iron levels.^12^ Alternatively, due to the iron-dependent regulation of HERC2, one could also argue that the high spleen iron levels in C57BL/6J could contribute to increased HERC2 mediated proteasomal degradation of NCOA4.^10^ Despite the fact that increased spleen non-heme iron content is not correlated with significantly lower serum iron levels in the C57BL/6J substrain, the increment in splenic macrophage iron levels is a possible explanation for the mild increase in Hct and MCV (Table 1). While the abovementioned differences between substrains were observed in older mice (24 weeks of age) they seem to be restricted to males since analysis of females at 12 weeks of age showed no difference regarding spleen iron content or ferritin levels (data not shown).

Our study extends on reported phenotypic differences between C57BL/6 substrains by revealing a previously undescribed splenic high non-heme iron content in C57BL/6J mice. This high-iron phenotype is restricted to the spleen, since no differences were observed regarding hepatic (Table 1; SDC Fig. 1A, Supplemental Digital Content) or duodenal (data not shown) iron content. These data demonstrate that C57BL/6 substrains, specifically C57BL/6N and C57BL/6J, are not the same. Therefore, the description of control substrains and mutant genetic backgrounds needs to be highly accurate to avoid a lack of reproducibility associated with laboratory mouse research. Variation in iron-related parameters in these two models highlights that different wildtype substrains cannot be used interchangeably for (iron) research as well as the need for more comprehensive reporting procedures.

## Supplementary Material

Supplemental Digital Content
