# Supplementary material for: Iron-Related Parameters are Altered Between C57BL/6N and C57BL/6J Mus Musculus Wild-Type Substrains
Source: Hemasphere. 2019 Nov 9;3(6):e304. doi: 10.1097/HS9.0000000000000304 (PMC6924556; doi:10.1097/HS9.0000000000000304)
Supplement: Supplemental Digital Content [file hs9-3-e304-s001.docx]

**Supplemental Digital Content, Materials and Methods**

**Mice**

12 male mice on a pure C57BL/6J genetic background were compared with 12 male mice on a pure C57BL/6N genetic background (colony founder mice purchased from Charles River). Mice were phenotyped for this study, in parallel, 6 generations following purchase. All mice were maintained and analyzed under absolutely identical conditions. Mice were 12-weeks-old and born and housed in the Heidelberg University animal facility under constant light-dark cycle and maintained on a standard mouse diet containing 200 ppm iron (LASQCdiet Rod 18-A – LASvendi) with *ad libitum* access to food and water. Mouse breeding and animal experiments were approved and conducted in compliance with the guidelines of the University of Heidelberg.

**Hematological and serum iron parameters**

Blood was collected in heparin tubes for evaluation of hematological parameters and in tubes with a gel clot activator for serum separation. Hematological parameters were determined using the scil Vet ABC hematology analyzer (ABX Diagnostics). Serum iron concentration and unsaturated iron binding capacity were determined using the SFBC and UIBC kits (Biolabo) following manufacturer’s instructions. Transferrin saturation was calculated using the formula SFBC / (SFBC + UIBC) x 100.

**Tissue iron measurements**

Liver, spleen and duodenum non-heme iron content was measured using the bathophenantroline method and calculated against dry weight tissue.[^1^](#_ENREF_1)

**DAB-enhanced Perls’ stain**

Tissues were fixed for 24 hours at 4°C by immersion in a solution of 10% neutral buffered formalin (Sigma), dehydrated and paraffin embedded. Tissues were sectioned at 3 μm and mounted on polysine slides (Thermo Scientific). Tissue sections were rehydrated and stained for 15 min with a potassium ferrocyanide/HCl solution (Sigma). After washing with distilled water, slides were treated with 3,3-diaminobenzidinetetrahydrochloride (DAB) (Sigma Aldrich), washed with distilled water and counterstained with hematoxylin (Sigma). Images were digitally acquired with a Nikon Ni-E microscope, using the Nikon NIS-Elements software.

**BMDM isolation and *Bmpr2* siRNA-mediated knockdown**

Bone marrow cells were flushed from femurs using cold PBS and filtered through a 40 µm cell strainer. Cells were seeded at a density of 350.000 cells/mL in RPMI1640-Glutamax medium (Life Technologies) supplemented with 10% FBS (Life Technologies), 1% penicillin/streptomycin (Sigma-Aldrich) and 10 ng/ml M-CSF (Peprotech). After 4 days, non-adherent cells were removed by washing with PBS and the medium was replaced daily. On the 5^th^ day, siBmpr2 (Silencer Select siRNA s63049, Ambion) transfection was performed with Lipofectamine RNAiMAX Reagent according to manufacturer’s recommendations. After 48 h, cells were collected for total RNA and protein extraction.

**RNA extraction, Reverse Transcription and qRT-PCR**

Total RNA was isolated from tissues using TRIzol (Life Technologies) and from BMDMs using the RNeasyPlus Mini kit (Qiagen) according to the manufacturer’s instructions. 1 or 0.5 μg of total RNA (tissues or BMDMs, respectively) was reverse transcribed in a 25 μL reaction mixture using RevertAid H Minus reverse transcriptase (Thermo Scientific) and random oligomers as primers. SYBR green qRT-PCR was performed using the StepONE Plus real-time PCR system (Applied Biosystems). mRNA expression of the gene of interest was normalized to *Rpl19* and data were analyzed using the ΔΔCt method.[^2^](#_ENREF_2) The primers used are listed in SDC, Table 1.

**Western Blotting**

Protein lysates were obtained by homogenizing snap-frozen tissues or cells in RIPA buffer supplemented with a protease inhibitor cocktail (cOmplete Tablets EASYpack, Roche). Protein concentration was determined using the DC protein assay. 50 μg of total protein extracts were separated by 10-12% SDS-PAGE and subjected to western-blot analysis using the antibodies listed in SDC, Table 2. Western blot images were acquired and quantified with the Vilber Lourmat Fusion-FX Chemiluminescence system. Vinculin and Actin were used as loading controls.

**Statistical Analyses**

Data are shown as mean ± standard error of the mean (SEM). Number of mice analyzed (n) is indicated in the figure legend. Statistical analyses were performed using Prism v6 (GraphPad Software). Comparisons between the two groups were performed using the two-tailed, Student’s t-test and p-values <0.05 (*), <0.01 (**), <0.001 (***) and <0.0001 (****) are indicated.

***In silico* modeling and mutagenesis of the Cytochrome b5 heme-binding domain structure of C57BL/6N Herc2**

Cytochrome b5 heme-binding domain structure of C57BL/6N HERC2 (sp|Q4U2R1|1208-1284) was modeled by using the SWISS-MODEL server (<https://swissmodel.expasy.org/>).[^3-7^](#_ENREF_3) The NMR structure of the homologous domain of human HERC2 (PDB name: 2KEO - identity = 92.21%) was used as a template. *In silico* mutagenesis (G1235D) and electrostatic surface potential analysis of the mouse HERC2 model were performed with PyMOL Molecular Graphics System. Interspecies alignment for the HERC2 Cytochrome b5 heme-binding domain protein sequence was performed with Clustal Omega (<https://www.ebi.ac.uk/Tools/msa/clustalo/>).

**Data availability**

The datasets generated and/or analyzed during the current study are available from the corresponding author on reasonable request.

**SDC, Figures**


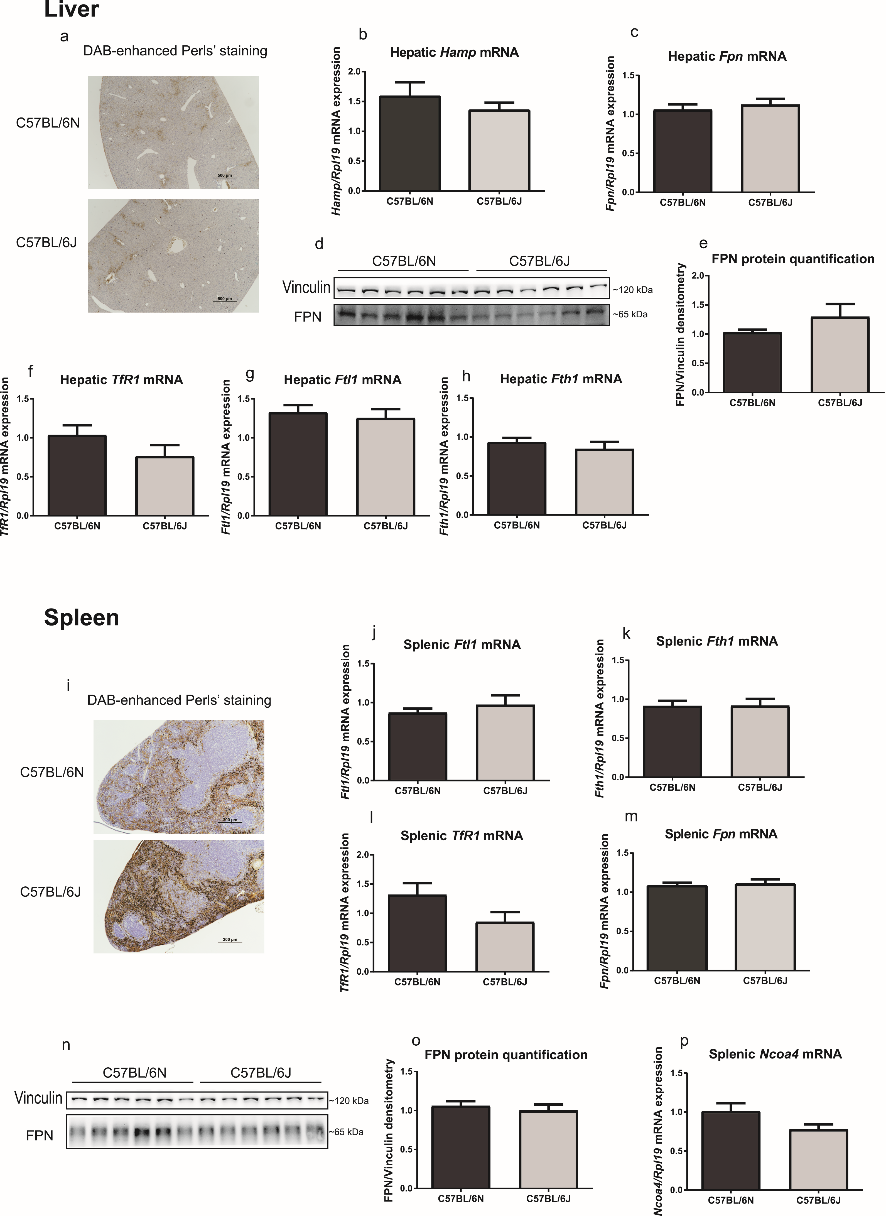


SDC, Figure 1. Additional iron-related parameters in liver and spleen. Liver: (a) Representative DAB-enhanced Perls’ stainings of livers of 12-week-old C57BL/6J and C57BL/6N mice. (b) qRT-PCR analysis of Hamp mRNA levels (n=12). (c) qRT-PCR (n=12) and (d,e) Western-blot (n=6) analysis of liver Fpn levels. (f) qRT-PCR analysis of *TfR1* mRNA levels (n=12). (g) qRT-PCR analysis of *Ftl1* mRNA levels (n=12). (h) qRT-PCR analysis of *Fth1* mRNA levels (n=12). Spleen: (i) Representative DAB-enhanced Perls’ stainings of spleens of 12-week-old C57BL/6J and C57BL/6N mice. (j) qRT-PCR analysis of *Ftl1* mRNA levels. (k) qRT-PCR analysis of *Fth1* mRNA levels. (l) qRT-PCR analysis of *TfR1* mRNA levels. (m) qRT-PCR (n=12) and (n,o) Western-blot (n=6) analysis of spleen Fpn levels. (p) qRT-PCR analysis of *Ncoa4* spleen mRNA levels. *Rpl19* was used to normalize qRT-PCR data and vinculin as a loading control for Western-blots. Data are reported as mean ± SEM.


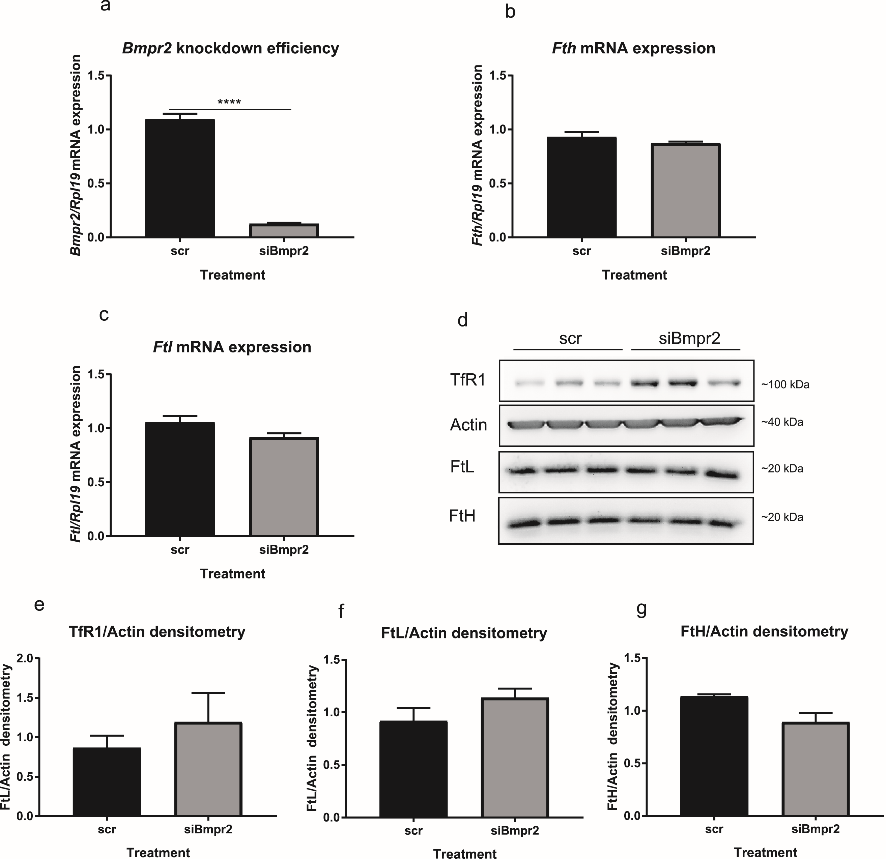
SDC, Figure 2. *Bmpr2* silencing does not affect Ferritin levels in BMDMs. (a) qRT-PCR analysis of *Bmpr2* mRNA levels after *Bmpr2* silencing in BMDMs (n=3). (b) qRT-PCR analysis of *Fth* mRNA levels after *Bmpr2* silencing in BMDMs (n=3). (c) qRT-PCR analysis of *Ftl* mRNA levels after *Bmpr2* silencing in BMDMs (n=3). (d-g) Western-blot analysis of TFR1 (d, e), FTL (d, f) and FTH (d, g) protein levels in BMDMs after *Bmpr2* silencing in BMDMs (n=3). *Rpl19* was used to normalize qRT-PCR data and actin as a loading control for Western-blots. One representative loading control is shown. Data are reported as mean ± SEM. Student’s t test: *p < 0.05; **p < 0.01; ***p < 0.001; ****p < 0.0001.


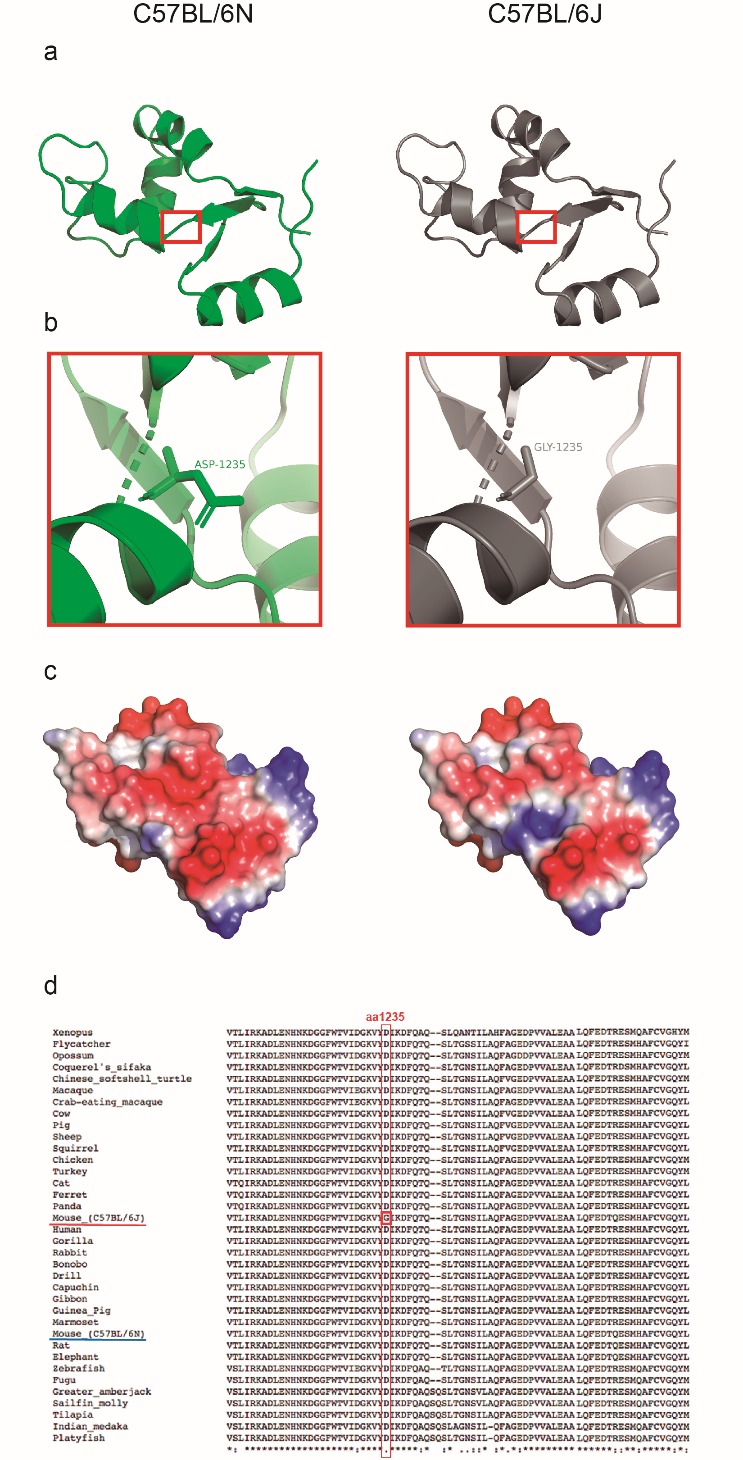


SDC, Figure 3. HERC2 missense mutation G1235D alters the electrostatic surface potential of the Cytochrome b5 heme-binding subdomain. (a) Secondary structure representation of the HERC2 Cytochrome b5 heme-binding subdomain encoded by C57BL/6N (left panel) and C57BL/6J (right panel). (b) High magnification of the missense mutation G1235D in the Cytochrome b5 heme-binding domain of HERC2. (c) Model of the electrostatic surface potential of the HERC2 subdomain of C57BL6/N (left panel) and C57BL6/J (right panel) mice. Red, blue and white areas represent negative, positive and neutral exposed charges, respectively. (d) Interspecies alignment of the HERC2 Cytochrome b5 heme-binding subdomain protein sequence. In the red square aa1235 for C57BL/6J is highlighted

**SDC, References**

1. Torrance JD, Bothwell TH. A simple technique for measuring storage iron concentrations in formalinised liver samples. *S. Afr. J. Med. Sci.* Apr 1968;33(1):9-11.

2. Pfaffl MW. A new mathematical model for relative quantification in real-time RT-PCR. *Nucleic Acids Res.* May 1 2001;29(9):e45.

3. Waterhouse A, Bertoni M, Bienert S, et al. SWISS-MODEL: homology modelling of protein structures and complexes. *Nucleic Acids Res.* Jul 2 2018;46(W1):W296-W303.

4. Bienert S, Waterhouse A, de Beer TA, et al. The SWISS-MODEL Repository-new features and functionality. *Nucleic Acids Res.* Jan 4 2017;45(D1):D313-D319.

5. Guex N, Peitsch MC, Schwede T. Automated comparative protein structure modeling with SWISS-MODEL and Swiss-PdbViewer: a historical perspective. *Electrophoresis.* Jun 2009;30 Suppl 1:S162-173.

6. Benkert P, Biasini M, Schwede T. Toward the estimation of the absolute quality of individual protein structure models. *Bioinformatics.* Feb 1 2011;27(3):343-350.

7. Bertoni M, Kiefer F, Biasini M, Bordoli L, Schwede T. Modeling protein quaternary structure of homo- and hetero-oligomers beyond binary interactions by homology. *Scientific reports.* Sep 5 2017;7(1):10480.

**Disclosures**

The authors declare no competing interests.

**Author Contributions**

O.M., J.N. and M.U.M. conceived the study. O.M., J.N., N.H., S.C. and C.G. performed experiments. O.M., J.N., N.H. and S.C. analyzed data. All authors interpreted the results and discussed their relevance. O.M. wrote the main manuscript text, which received comments and corrections from all authors. M.U.M. supervised the study. All authors reviewed the manuscript.

**SDC, Tables**

| SDC, Table 1. SYBR-green qRT-PCR primers. | | |
| --- | --- | --- |
|  | Primer Forward | Primer Reverse |
| *Rpl19* | AGGCATATGGGCATAGGGAAGAG | TTGACCTTCAGGTACAGGCTGTG |
| *Fpn* | TGTCAGCCTGCTGTTTGCAGGA | TCTTGCAGCAACTGTGTCACCG |
| *TfR1* | CCCATGACGTTGAATTGAACCT | GTAGTCTCCACGAGCGGAATA |
| *Hepcidin* | ATACCAATGCAGAAGAGAAGG | AACAGATACCACACTGGGAA |
| *Bmpr2* | GAGCACAGAGGCCCAATTCT | CATCTTGTGTTGACTCACCTACT |
| *FtL* | CGTGGATCTGTGTCTTGCTTC | GCGAAGAGACGGTGCAGACT |
| *FtH* | TGGAACTGCACAAACTGGCTACT | ATGGATTTCACCTGTTCACTCAG |
| *Ncoa4* | CTTTCGCCCCGACTGTTAC | CGATTCTGGTAGTCTGTTCCTTC |

| SDC, Table 2. Antibodies used for western blotting. | | | |
| --- | --- | --- | --- |
| Antigen | Host | Dilution | Reference |
| FPN | Rabbit | 1:500 | MTP11-A / Alphadiagnostics |
| TfR1 | Mouse | 1:1000 | 136800 / Invitrogen |
| FtL | Rabbit | 1:2500 | ab69090 / Abcam |
| FtH | Rabbit | 1:1000 | ab183781 / Abcam |
| Vinculin | Mouse | 1:1000 | V9131 / Sigma-Aldrich |
| Actin | Mouse | 1:10000 | A1978 / Sigma-Aldrich |
| NCOA4 | Rabbit | 1:1000 | the NCOA4 antibody was kindly provided by Dr. Francesca Carlomagno |
